# Inebriate Asylums

**Published:** 1874-08

**Authors:** 


### INEBRIATE ASYLUMS.

A SORROWFUL tale comes from Binghamton, where the State of New York, with her characteristic liberality, has expended a large amount of money in erecting buildings to afford facilities for recovering persons from the habit of getting drunk. It is reported that Dr. Bush, the chaplain of the asylum, in retiring from his office, has given it as his opinion, that, practically, the institution is a failure. That of the eighty-two patients in the establishment, only three are even apparently cured, and these three are intelligent, well educated men, from the higher walks of life. One of the menials of the establishment, who seemed to have belonged to a higher station in life, and to have been a close observer of the workings of the institution for a number of years, said, "There is no cure except in keeping it out of their reach." There is more truth in that one sentence than in all the volumes ever written on the subject. A truth which tells more—a truth most terribly suggestive. Now and then a man who has been a drunkard breaks off from the habit and goes down to the grave without even touching another drop; but such a man does it by a force of will not possessed by any two in a million. The only other persons who cease to get drunk, after having been in the habit of it, are either those who are shut up in a prison or penitentiary, where it is impossible to get it—and these are cured for no longer a time than they obtain their liberty—or those who secretly use some other form of stimulant. Some of the humane and tender hearted assert, and with considerable scientific show of logic, that drunkenness is a disease. But we fail to see how that helps the matter; it is not the less a calamity and a curse. Disease though it be, it is one self-brought, and is so much the more utterly inexcusable—and the stronger the reason, the louder the warning against ever touching a drop of the accursed thing, until three score has been reached, unless advised by a physician. When that is the case, just toss the doctor and the dose out of the window.

